# Supplementary figures and images for: Divergence between Hemichannel and Gap Junction Permeabilities of Connexin 30 and 26
Source: Life (Basel). 2023 Jan 31;13(2):390. doi: 10.3390/life13020390 (PMC9962233; doi:10.3390/life13020390)

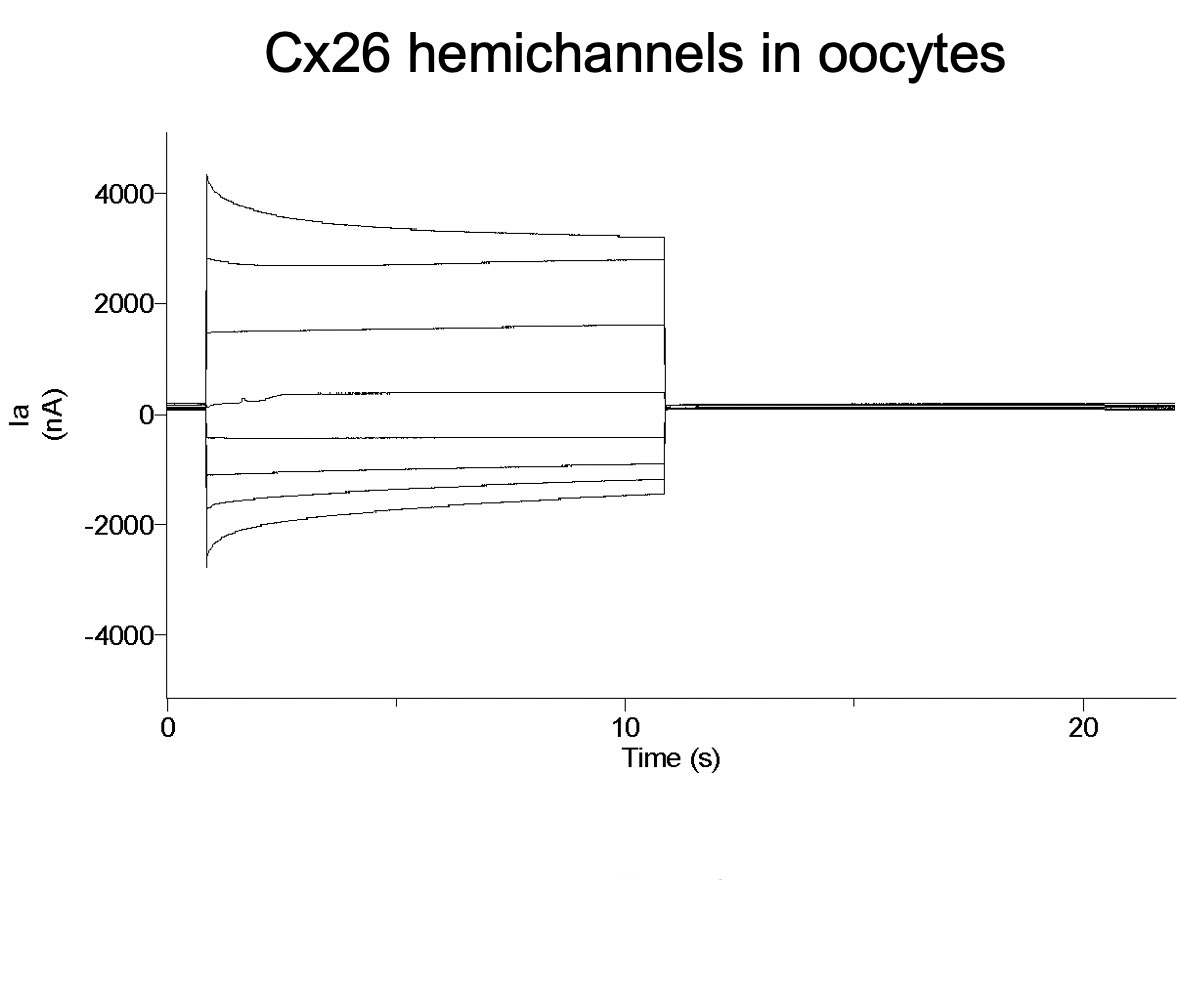

Supplement: Supplementary file 1 [file life-13-00390-s001.zip › Figure S1.jpg]
